# Dynamic Task Allocation in Multi-Robot System Based on a Team Competition Model

**DOI:** 10.3389/fnbot.2021.674949

**Published:** 2021-05-20

**Authors:** Kai Jin, Pingzhong Tang, Shiteng Chen, Jianqing Peng

**Affiliations:** ^1^Department of Computer Science and Engineering, The Hong Kong University of Science and Technology (HKUST), Hong Kong, China; ^2^Institute for Interdisciplinary Information Sciences, Tsinghua University, Beijing, China; ^3^Institute of Software, Chinese Academy of Sciences, Beijing, China; ^4^School of Intelligent Systems Engineering, Sun Yat-sen University, Shenzhen, China

**Keywords:** team competition, task allocation, multi-robot system, dominated players, sub-game perfect equilibrium

## Abstract

In recent years, it is a trend to integrate the ideas in game theory into the research of multi-robot system. In this paper, a team-competition model is proposed to solve a dynamic multi-robot task allocation problem. The allocation problem asks how to assign tasks to robots such that the most suitable robot is selected to execute the most appropriate task, which arises in many real-life applications. To be specific, we study multi-round team competitions between two teams, where each team selects one of its players simultaneously in each round and each player can play at most once, which defines an extensive-form game with perfect recall. We also study a common variant where one team always selects its player before the other team in each round. Regarding the robots as the players in the first team and the tasks as the players in the second team, the sub-game perfect strategy of the first team computed via solving the team competition gives us a solution for allocating the tasks to the robots—it specifies how to select the robot (according to some probability distribution if the two teams move simultaneously) to execute the upcoming task in each round, based on the results of the matches in the previous rounds. Throughout this paper, many properties of the sub-game perfect equilibria of the team competition game are proved. We first show that uniformly random strategy is a sub-game perfect equilibrium strategy for both teams when there are no redundant players. Secondly, a team can safely abandon its weak players if it has redundant players and the strength of players is transitive. We then focus on the more interesting case where there are redundant players and the strength of players is not transitive. In this case, we obtain several counterintuitive results. For example, a player might help improve the payoff of its team, even if it is dominated by the entire other team. We also study the extent to which the dominated players can increase the payoff. Very similar results hold for the aforementioned variant where the two teams take actions in turn.

## 1. Introduction

In the past two decades, intelligent multi-robot systems are more and more widely used in industrial manufacturing, agriculture, hospital, fire rescue, cargo handling, entertainment, and many other places. The efficiency of the systems is crucial to their applications and it highly depends on the collaboration between robots. One of the primary problem that occurs to the designer of multi-robot systems is how to assign tasks to robots such that the most suitable robot is selected to execute the most appropriate task, which is usually referred to as the *task allocation problem* and which arises in all kinds of real-life applications.

It is well-known that game theory lays the mathematical foundation for the research of collaboration in multi-robot system, and it is a trend to integrate the ideas and theoretical results in game theory into the research of multi-robot system. For example, market-based approaches to task allocation are proposed in Botelho and Alami (1999), Gerkey and Mataric (2002), Wang et al. (2004), Dias et al. (2006), Zlot and Stentz (2006), and Wu and Shang (2020). In this paper, a team-competition model, which is of interest by itself in game theory, is proposed to solve a Dynamic Multi-Robot Task Allocation (DMRTA) problem.

In our DMRTA problem, there are *m* robots and *n* pre-described tasks, and the tasks are coming in *T* rounds, where *T* ≤ min{*m, n*}. One task will come in every round, and it would like to be assigned to one robot immediately. Be aware that when *T* < *n*, only *T* tasks, but not all the *n* tasks, will be assigned (as there are only *T* rounds), and this means the set of tasks to be assigned are not fully determined at the beginning in our problem. For simplicity, assume that each pre-described task comes at most once (this constraint can easily be removed by replicate the task) and that each robot can take at most one task (as well, this constraint can be removed by replicate the robot). Different robots have different performances in solving different tasks, and to describe this diversity we make the following assumption: if the robot with index *r* (i.e., robot *r*) is to execute the task with index *s* (i.e., task *s*), there is a probability *p*_*r, s*_ that robot *r* succeeds to complete its job—and a probability 1 − *p*_*r, s*_ that it fails to do its job. The *m* × *n* probability matrix *p*_*r, s*_ ∣ 1 ≤ *r* ≤ *m*, 1 ≤ *s* ≤ *n* is prior information—it is given to us before the allocation mission starts. Roughly, our objective is that the number of successful robots is as high as possible—in other words, the number of tasks that have been solved successfully is as high as possible. Details of the DMRTA problem will be elaborated right after we introduce our team competition model in what follows.^1^

We investigate a type of *team competitions* where there are two teams, each with a number of players, competing against each other. The competition proceeds in a fixed number of rounds. In each round, each team simultaneously sends out a player to a match (We also consider a variant where one team, say Team 2 without loss of generality, always takes actions before the other team in each round. This variant will mainly be discussed in section 3). The result of the match is then revealed according to a probabilistic strength matrix between players. The selected players cannot compete in the subsequent rounds. The competition proceeds to the next round if there is one; or terminated otherwise. The format of the competition and the strength matrix are common knowledge to both teams. The final payoff of each team is the number of matches it wins. To make it more general, we also investigate another commonly seen form where each team gets payoff 1 if it wins strictly more matches than the other team, 0 if ties, and −1 if it wins less matches. Clearly, this competition between two teams defines a standard *extensive-form game*, or more precisely, a *stacked matrix game* (Lanctot et al., 2014). We are interested in the sub-game perfect equilibria of the game, i.e., a strategy profile that specifies for each team which player to play at each round. A formal description of our team competition model is given in the next section.

Our team competition model is first motivated by the Chinese horse race story described in Tang et al. (2009) (see also Wikipedia, 2015b). It represents one of the most popular forms of horse races where each team ranks its horses to match sequentially. Moreover, the Swaythling Cup, as known as World Table Tennis Championships, follows the same model described in our paper: each team adaptively selects a ranking of three players and brings two additional substitutes. In fact, this has been one of the most popular formats of team competition in table tennis. In addition, the card game Goofspeil (Lanctot et al., 2014; Wikipedia, 2015a) also falls into nearly the same model as described in our paper. Last but not least, many military engagements (like fighting between two groups of drones) may also have this type of structure.

Obviously, we can regard the *m* robots as the players in the first team and the *n* tasks as the players in the second team, and the probability matrix *p*_*r, s*_ ∣ 1 ≤ *r* ≤ *m*, 1 ≤ *s* ≤ *n* can serve as the probabilistic strength matrix in the team competition. Then, the (sub-game perfect) strategy profile of the first team we obtained via solving the team competition gives us a solution for allocating the tasks to the robots—it specifies how to select the robot (randomly according to some probability distribution given by the strategy) to execute the upcoming task in each round, based on the results of the matches in the previous rounds.

At this place, it is necessary to point out a feature of our DMRTA problem inherited from the team competition model: As the two teams shall send out their players simultaneously in each round, in our DMRTA problem we shall select the robot before the task in the corresponding round is revealed to us. Nevertheless, if we would like to handle the case where we can select the robot after the task in the corresponding round is revealed, we only need to deal with the variant where Team 2 reacts before Team 1. Gladly, we will see in section 3 that many results for this non-simultaneous variant are aligned with the results for the original simultaneous case (In particular, most of the results are the same. See Table 1 for a comparison). We first discuss the simultaneous case because this case is typical and more difficult.

**Table 1:** Summarize of the results for simultaneous case and non-simultaneous case.

	**Simultaneous case**	**Non-simultaneous case**
*n* = *m* = *T*	The value of the game equals the average utility (of Team 1) across all matchings. Moreover, the uniformly random strategy is a SPE strategy (Theorem 1).	The value equals the maximal utility that Team 1 can gain over all matchings. The pure strategy ensuring the best matching for Team 1 is a SPE strategy (Theorem 5).
Transitive strength	If *A*_*m*_ ≤ … ≤ *A*_1_, *A*_*T*+1_, …, *A*_*m*_ can be removed. If *B*_*n*_ ≤ … ≤ *B*_1_, *B*_*T*+1_, …, *B*_*n*_ can be removed (Theorem 2).	Exactly the same result holds (Theorem 6).
For non-transitive strength: *When other players are weaker than the first *T* players in a team, can the weaker players be removed?*	For *U* = *U*_*E*_, the answer is YES as long as *n* = *T*. For other utility functions *U* (e.g., *U*_*M*_), the answer is NO (Theorem 3).	For Team 1, the answer is YES as long as *n* = *T* (this holds for any *U*). However, the answer is NO for Team 2 (Theorem 7).
For non-transitive strength: *How many dominated players shall we recruit to achieve maximum value?*	$\{\begin{array}{cc} T-1 & \text{for }U=U_{E};\\ \left\lfloor T/2\right\rfloor & \text{for }U=U_{M}. \end{array}$ (Theorem 4)	Exactly the same result holds (Theorem 8).

Despite the aforementioned application in the DMRTA problem, our team competition model is interesting by itself in game theory. We are particularly interested in a situation where at least one team has more players than the number of rounds in the competition. As a result, some players will never have chance to participate in any match. A main agenda of this paper is to understand to what extent can the presence of additional players affect the payoff of both teams. In particular, we ask the following questions: (1) Can the presence of additional weakest teammate, a teammate whose row in the strength matrix is strictly dominated by any other row, help increase the payoff of the team? (2) Can the presence of additional dominated teammate, a teammate that always loses to any player in the opponent team, help increase the payoff of the team? It might appear intuitive that the answers to both questions are negative. For the first question, it seems that the weakest teammate will never have a chance to participate in any match since one can always replace him by a better teammate and increase payoff. For the second question, it might seem more obvious since the dominated teammate will lose any matches thus must be replaced by a better teammate. To our surprise, we find that the answers to both questions are affirmative.

Our contributions to the team competition model are summarized in the following. We first show that uniformly random strategy is a sub-game perfect equilibrium strategy for both teams when there are no redundant players (i.e., the number of players in each team equals the number of rounds). The uniformly random strategy always picks the unmatched player uniformly at random in each round. Then, we consider the general case where at least one team has redundant players. We first study the case where the strength of players is transitive (see Definition 2), which means that the players can be rearranged in a queue so that each of them is weaker than its successor. We prove that, a team can safely abandon its weak players if it has redundant players and the strength of players is transitive. Therefore, this case reduces to the case where there are no redundant players. Finally, we focus on the case where there are redundant players and the strength of players is not transitive. In this case, we obtain a number of counterintuitive results. Most importantly, a player might help improve its team's payoff, even if it is dominated by the entire opposing team. We give a necessary condition for a dominated player to be useful, which alternatively suggest that a particular utility function (named *U*_*E*_ below) is more reasonable in team competition. Our results imply that a team can increase its utility by recruiting additional dominated players. We further show that, the optimal number of dominated players to recruit can scale with the number of rounds. More precisely, this number can be Θ(*T*) if there are *T* rounds. Last but not least, we study the limitation of dominated players. These results bring insights into playing and designing general team competitions.

Team competition has been studied for years. Tang et al. (2009, 2010) study a team competition setting where the number of players equals the number of rounds and both teams must determine the ordering of players upfront, before the competition starts. They put forward competition rules that are truthful while satisfy other desirable properties. The main difference between their work and ours is that we do not design new mechanisms but study game theoretical properties of commonly used competition rules. The differences also lie in that the strategies are adaptive in our setting and each team can have more players than the rounds. The strategic aspects of team competition have also been under scrutiny of computer scientists due to a recent Olympic scandal in badminton, where several teams deliberately throw matches in order to avoid a strong opponent in the next round. The phenomenon has been discussed in depth in a series of algorithmic game theory blogposts by Kleinberg (2012) and Procaccia (2013). A parallel literature has been concerned with the strategic aspect of tournament seeding (Hwang, 1982; Rosen, 1986; Knuth, 1987; Schwenk, 2000; Altman et al., 2009; Vu et al., 2009). It is well-known that there are cases where by strategic seeding and structuring, any player can be winner in knockout tournament. Various game theoretical questions, such as player-optimal seeding, complexity of manipulation and incentives to guarantee strategyproofness, have been investigated in this literature.

The study of various kinds of multi-robot task allocation problem using game theory dates back to early 2000's. Botelho and Alami (1999), Gerkey and Mataric (2002), Wang et al. (2004), Dias et al. (2006), Zlot and Stentz (2006), and Wu and Shang (2020) make use of the theory of market economies to determine how to allow robots to negotiate on responsibilities in task allocation. In particular, they discuss how to manage bids (robots communicate to bid for tasks according to their expected contribution to the tasks), how to handle bids in parallel and how to handle multiple tasks at once, and so on. Usually, heuristic assignments are made by assigning every task to the robot that can execute it with the highest utility.

## 2. Materials and Methods

Team 1 has a set of *m* players {*A*_1_, …, *A*_*m*_}. Team 2 has a set of *n* players {*B*_1_, …, *B*_*n*_}. A competition between team 1 and 2 is a tuple *G*(*T, P, U*) where,

1. *T* is the number of rounds.
2. In each round, each team **simultaneously** selects one of its players that have not been selected yet.
3. *P* is a probabilistic matrix that describes the relative strength between players, with *P*_*i, j*_ denoting the probability that *A*_*i*_ wins against *B*_*j*_ and 1 − *P*_*i, j*_ the probability for *A*_*i*_ to lose to *B*_*j*_.
4. *U*:[*T*] → *R* denotes the utility function of each team. The utility function only depends on the number of rounds *t* each team wins, i.e., it can be represented by *U*(*t*). This also implies that both teams have the same utility functions.
5. The parameters *n, m, T, P, U* are common knowledge to both teams, and historical plays are perfectly observable. It is assured that *P*_*i, j*_ ∈ [0, 1] for all *i, j* and that *m* ≥ *T* and *n* ≥ *T* so that there are enough players to complete the competition.

The following utility functions *U*_*E*_ and *U*_*M*_ are two commonly seen ones:

(1) $$\begin{array}{r} U_{E}\left(t\right)=t-T/2.\;U_{M}\left(t\right)=\{\begin{array}{cc} 1 & t>T/2\\ 0 & t=T/2\\ -1 & t<T/2 \end{array}. \end{array}$$

In other words, *U*_*E*_ describes a competition where a team's utility is exactly the number of rounds it wins (minus some constant *T*/2); while *U*_*M*_(*t*) describes a competition where a team's utility is whether it wins more than its opponent. Notice that, when *U* = *U*_*E*_ or *U* = *U*_*M*_, we have *U*(*t*) + *U*(*T* − *t*) = 0, hence both utility functions define a zero-sum game. In this paper we always assume that *U*(*T*) + *U*(*T* − *t*) = 0.

### 2.1. Example: Simultaneous Card Games

The models above formulates the standard team competitions as commonly seen under the context of sports, but shall not be limited to sports. The following is an instance of card games that fall into our framework. Suppose that Alice and Bob each has a deck of three cards. In each deck one card is in suit ♡ and two cards are in suit ♠. They play three rounds; in each round Alice and Bob select one card and they reveal the cards simultaneously. If they select cards in same suit (both in ♡ or both in ♠), Alice wins this round; otherwise Bob wins this round. The one who wins two or three rounds gets utility 1; the other one wins zero or one rounds and it gets utility −1.

This game can be conveniently represented in our model using the following parameters:

$$\begin{array}{l} m=n=T=3,P=\left(\begin{array}{ccc} 1 & 0 & 0\\ 0 & 1 & 1\\ 0 & 1 & 1 \end{array}\right),U=U_{M}. \end{array}$$

For this game, applying our first theorem, a (sub-game perfect) equilibrium strategy for both players exists, and it is to just to play uniformly random. It follows that Alice and Bob have utilities −1/3 and +1/3.

### 2.2. Extensive-Form Game With Perfect Recall

Any particular instance *G*(*T, P, U*) of our team competition is an extensive-form game. In this game, a history can be described by a tuple (*k*, **a**, **b**, **c**) where:

- *k* indicates the number of rounds that has been played;
- **a** is a *k*-dimensional vector which stores the players selected by Team 1 in the past *k* rounds;
- **b** stores the players selected by Team 2;
- **c** is a *k*-dimensional 0–1 vector which stores the results in the first *k* rounds, where 0 corresponds to a lose by Team 1 and 1 corresponds to a win by Team 1.

A behavioral strategy in this game is a mapping from every history to a probability distribution over actions. That is, at each history, the strategy of each team is to pick the next player according to a probability distribution. By Kuhn' Theorem (Kuhn, 1953; Osborne and Rubinstein, 1994), there is a sub-game perfect equilibrium (SPE), in which both teams use behavioral strategies. According to the SPE, a value *V*(*H*) can be defined for each history *H*, which indicates the expected utility that Team 1 would get at the end of the game if it is now at history *H*. Note that each history is the root of a sub-game and so the value of a history is the same as the value of the sub-game.

### 2.3. Computing SPE

By backward induction, one can easily get

**Lemma 1**. *Two histories have the same value if they have selected the same players to play (but may be in different orders) and Team 1 won the same number of rounds*.

Based on the above lemma, the histories can be partitioned to equivalence classes, such that each equivalence class corresponds to a four-tuple (*k, X, Y, w*): *k* is a number in [*T*] which denotes the number of past rounds; *X* is a subset of *A* of size *k*; *Y* is a subset of *B* of size *k*; *X, Y* denote the players that have played; *w* is a number in [*k*] which denotes how many rounds Team 1 has won so far.

In the following, we show in detail how to compute the value of each equivalence class via dynamic programming.

Let *V*[*k, X, Y, w*] denote the expected utility of Team 1 when the history belongs to class (*k, X, Y, w*).

Clearly, we have *V*[*k, X, Y, w*] = *U*(*w*) when *k* = *T*.

When *k* < *T*, computing *V*[*k, X, Y, w*] reduces to computing the value of the matrix game *M*(*k, X, Y, w*) where the matrix *M*(*k, X, Y, w*) is defined as follows:

It consists of *m* − *k* rows and *n* − *k* columns. Each row corresponds to a player in *A* − *X*, and each column corresponds to a player in *B* − *Y*. The cell corresponding to *A*_*i*_, *B*_*j*_ equals to the expect utility of Team 1 when Team 1 and Team 2, respectively make action *A*_*i*_ and *B*_*j*_ on the current state (*k, X, Y, w*), which equals

$$\begin{array}{l} \begin{array}{c} V\left[k+1,X+\left\{A_{i}\right\},Y+\left\{B_{j}\right\},w+1\right]\cdot P_{i,j}+\\ V\left[k+1,X+\left\{A_{i}\right\},Y+\left\{B_{j}\right\},w\right]\cdot \left(1-P_{i,j}\right). \end{array} \end{array}$$

The reason behind the above definition of *M*(*k, X, Y, w*) is as follows. If the two teams select *A*_*i*_, *B*_*j*_ in this round, Team 1 has probability *P*_*i, j*_ to win this round and hence the history becomes (*k* + 1, *X* + {*A*_*i*_}, *Y* + {*B*_*j*_}, *w* + 1); besides, Team 1 has probability (1 − *P*_*i, j*_) to lose this round and hence the history becomes (*k* + 1, *X* + {*A*_*i*_}, *Y* + {*B*_*j*_}, *w*).

We can compute the value of all equivalent classes of histories according to the above induction. In fact, by computing these values, we also find a sub-game perfect behavior strategy for both players. To see this, suppose that (*k, X, Y, w*) is a non-terminal equivalent class of history. On solving the matrix game *M*[*k, X, Y, w*] we find the strategies for all the histories in the history class (*k, X, Y, w*).

### 2.4. Uniformly Random Strategies

The next theorem states that, if there are no redundant players, uniformly random is an equilibrium strategy for the teams. It holds for arbitrary utility function including *U*_*E*_ and *U*_*M*_.

**Definition 1**. *The* uniformly random strategy *is a behavioral strategy, in which a team always selects from the remaining players uniformly at random in each round*.

**Theorem 1**. *When both teams have no redundant players (i.e., n = m = T), then it is a SPE when both teams apply the uniformly random strategy*.^2^

We apply the following lemma for proving Theorem 1.

**Lemma 2**. *Suppose that there are no redundant players. Let* 𝕊 *denote the set of all perfect matchings between* {*A*_1_, …, *A*_*T*_} *and* {*B*_1_, …, *B*_*T*_}. *If Team 1 **or** Team 2 applies the uniformly random strategy, then the probability that the competition ends with any fixed matching in* 𝕊 *is exactly* 1/(*T*!).

Proof of Lemma 2: We only prove that the statement holds when Team 1 applies the uniformly random strategy. Symmetrically, the statement holds when Team 2 applies the uniformly random strategy.

First, suppose that Team 1 applies the uniformly random strategy while Team 2 applies an arbitrary pure strategy.^3^ In this case, we claim that the probability that the competition ends with any fixed matching is exactly 1/(*T*!). This can be proved by induction on the number of remaining τ rounds. In the stage with τ remaining rounds, let *M* be any fixed matching between the τ unused players in Team 1 and the τ unused players in Team 2. In the next round, it occurs with probability 1/τ that some edge *e* of *M* is chosen, because Team 1 will assign the player *B*_*i*_ selected by Team 2 to a random player among the unused players in Team 1. If this occurs, denote by *M*′ = *M* − {*e*} the matching obtained by deleting *e* from *M*, which is chosen with probability 1/((τ − 1)!) by induction hypothesis. Thus, *M* is chosen with probability 1/(τ!).

Finally, since a mixed strategy is a linear combination of the pure strategies, our job is done.    □

Proof of Theorem 1: Let 𝕊 be the same set as in Lemma 2. As there are no redundant players, a game will always end with some matching in 𝕊. For any matching *s* ∈ 𝕊, let *Z*_*s*_ denote the event that the game ends with this matching. If Team 1 applies the uniformly random strategy, it has expected utility

$$\begin{array}{l} \underset{s\in S}{\sum }E\left(\text{the utility of Team 1}|Z_{s}\right)/\underset{\text{Team 1 uniformly random}}{\mathrm{Pr}}\left(Z_{s}\right)\\ =\underset{s\in S}{\sum }E\left(\text{the utility of Team 1}|Z_{s}\right)/\left(T!\right) \end{array}$$

The second equation is according to Lemma 2, which states ${\mathrm{Pr}}_{\text{Team 1 uniformly random}}\left(Z_{s}\right)=1/\left(T!\right)$.

Similarly, if Team 2 applies the uniformly random strategy, it will get

$$\begin{array}{l} \underset{s\in S}{\sum }E\left(\text{the utility of Team 2}|Z_{s}\right)/\left(T!\right)\\ =\underset{s\in S}{\sum }-E\left(\text{the utility of Team 1}|Z_{s}\right)/\left(T!\right) \end{array}$$

Therefore, it is a Nash Equilibrium if both teams apply the uniformly random strategy. The argument can be similarly extended to show that it is SPE.    □

In the remainder of this paper, we focus on the case where there are redundant players.

**Claim 1**. *If there are redundant players, then the uniformly random strategy may not be a SPE strategy (For any team, a SPE strategy for this team requires that it is optimal in each subgame)*.

This claim is obvious for a team with redundant players; but less obvious for a team without redundant players. Here we give an example in which the uniformly random strategy is not a SPE strategy for a team with no redundant players.

**Example 1**. *Let m* = *T* = 2, *n* = 3, *U* = *U*_*E*_ = *U*_*M*_ (*U*_*E*_ = *U*_*M*_ *when* *T* = 2), $P=\left(\begin{array}{ccc} 0 & 0 & 1\\ 1 & 1 & 0 \end{array}\right)$.

According to method given in subsection 2.3, we can compute that $M(0,\emptyset ,\emptyset ,0)=\left(\begin{array}{ccc} -1 & -1 & 1\\ 0 & 0 & -1 \end{array}\right)$. Therefore, in the SPE, the behavior for Team 1 on the initial state should be select *A*_1_ with probability 1/3 and select *A*_2_ with probability 2/3. This guarantees (expected) utility −1/3. If to the contrary that Team 1 adopts the uniformly random strategy, it should select *A*_1_, *A*_2_ with probability 1/2, which would only guarantees (expected) utility −1/2.

### 2.5. Transitive Strength

**Definition 2**. *Player A_i_ is weaker than its teammate A_j_, denoted by A_i_ ≤ A_j_, if for any opponent B_k_, the probability of “A_i_ wins against B_k_” is less than or equal to the probability of “A_j_ wins against B_k_.” Similar for Team 2. Team 1 {A*_1_, …, *A_m_} are transitive if there is a permutation π of 1, …, m, such that A_π(1)_ ≤ … ≤ A_π(m)_. Similar for Team 2*.

**Definition 3**. *A utility function U is monotone if U*(*t* + 1) ≥ *U*(*t*) *for t* ∈ 0, … *T* − 1.

**Theorem 2**. *Assume monotone utility function. Then, (1) If A*_*m*_ ≤ … ≤ *A*_1_, *Team 1 has a SPE strategy which only selects, in each round, one of the players in A*_1_, …, *A*_*T*_. *(2) Symmetrically, if B*_*n*_ ≤ … ≤ *B*_1_, *Team 2 has a SPE strategy which only selects, in each round, one of the players in B*_1_, …, *B*_*T*_.

By combining Theorem 1 and Theorem 2, we can immediately get the following

**Corollary 1**. *When players in each team are transitive and U is monotone, there is a simple SPE strategy for both teams as follows. Assume that A*_*m*_ ≤ … ≤ *A*_1_ *and B*_*n*_ ≤ … ≤ *B*_1_. *Then, the SPE strategy for Team 1 is to select an unused player in A*_1_, …, *A*_*T*_ *uniformly random in each round; a SPE strategy for Team 2 is to select an unused player in B*_1_, …, *B*_*T*_ *uniformly random in each round*.

Theorem 2 and Corollary 1 have many applications. In the real word, the utility function is monotone, and in many situations, such as in board or sport games, it is indeed the case that the players are transitive.

We prove Theorem 2 (1) in the next; the claim (2) is symmetric.

We first provide two basic terminologies which are necessary for understanding the subsequent proof. Suppose that *A*_*m*_ ≤ … ≤ *A*_1_ and that *A*′ is a subset of *A* and $A^{\prime }=\left(A_{i\left[1\right]},\ldots A_{i\left[\left|A^{\prime }\right|\right]}\right)$, where *i*[1] < … < *i*[|*A*′|]. Then, for any 0 ≤ *C* ≤ |*A*′|, the top *C* players of *A*′ refers to {*A*_*i*[1]_, …, *A*_*i*[*C*]_}, and the rank *C* player of *A*′ refers to *A*_*i*[*C*]_.

**Lemma 3**. *Suppose that U*() *is monotone*.

1. *Consider a pair of history classes H*_1_ = (*k, X*_1_, *Y, w*_1_) *and H*_2_ = (*k, X*_2_, *Y, w*_2_). *We claim that, if the top T* − *k players of A − X*_1_ *and the top T − k players of A − X*_2_ *are the same and w*_1_ ≥ *w*_2_, *then V*(*H*_1_) ≥ *V*(*H*_2_).
2. *Let H* = (*k, X, Y, w*) *be a non-terminal history class. Let A*_*u*_ *be the rank T − k player in A* − *X and A*_*v*_ *be any player in A* − *X that is not a top T − k player. Then, the row in M*(*k, X, Y, w*) *that corresponds to A*_*u*_ *dominates the row that corresponds to A*_*v*_. *As a result, there is an equilibrium strategy at history H (for Team 1) which only selects the top T − k unmatched players to play*.

Proof. We prove it by backward induction. When *k* = *T*, Claim 1 holds according to the monotone property of *U*(); and Claim 2 naturally holds since it is a terminal history.

Now, we argue that, for 0 ≤ *k* < *T*, if the lemma holds for *k* + 1, it also holds for *k*.

First, we prove Claim 2. Let us compare the two rows corresponding to *A*_*u*_ and *A*_*v*_. Let us fix a column, say the one corresponding to *B*_*r*_. The cell corresponding to (*A*_*u*_, *B*_*r*_) is

$$\begin{array}{l} \begin{array}{c} M\left[u,r\right]=\underset{a}{\underset{︸}{V\left(k+1,X+\left\{A_{u}\right\},Y+\left\{B_{r}\right\},w+1\right)}}\cdot P_{u,r}\\ \;+\underset{b}{\underset{︸}{V\left(k+1,X+\left\{A_{u}\right\},Y+\left\{B_{r}\right\},w\right)}}\cdot \left(1-P_{u,r}\right) \end{array} \end{array}$$

The cell corresponding to (*A*_*v*_, *B*_*r*_) is

$$\begin{array}{l} \begin{array}{c} M\left[v,r\right]=\underset{a^{\prime }}{\underset{︸}{V\left(k+1,X+\left\{A_{v}\right\},Y+\left\{B_{r}\right\},w+1\right)}}\cdot P_{v,r}\\ \;+\underset{b^{\prime }}{\underset{︸}{V\left(k+1,X+\left\{A_{v}\right\},Y+\left\{B_{r}\right\},w\right)}}\cdot \left(1-P_{v,r}\right) \end{array} \end{array}$$

Notice that the top *T* − *k* − 1 players in *A* − *X* − {*A*_*u*_} and *A* − *X* − {*A*_*v*_} are the same. So, from the induction hypothesis, *a*′ ≥ *a* ≥ *a*′ ≥ *b* ≥ *b*′ ≥ *b*, i.e., *a* = *a*′ ≥ *b* = *b*′.

Since that *A*_*u*_ is the top *T* − *k* player while *A*_*v*_ is not, player *A*_*v*_ is weaker than *A*_*u*_, which means that *P*_*u, r*_ ≥ *P*_*v, r*_.

Combining the above arguments, we get that

$$\begin{array}{l} M\left[u,r\right]-M\left[v,r\right]=\left(a-b\right)\cdot \left(P_{u,r}-P_{v,r}\right)\geq 0. \end{array}$$

Therefore, *M*[*u, r*] ≥ *M*[*v, r*], and thus Claim 2 holds.

Then, we prove Claim 1. Let *M*_1_ denote *M*(*k, X*_1_, *Y, w*_1_) and *M*_2_ denote *M*(*k, X*_2_, *Y, w*_2_) for short. Suppose that *A*_*u*_ is a top *T* − *k* player in *A* − *X*_1_ (which is also a top *T* − *k* player in *A* − *X*_2_) and that *B*_*r*_ is any player in *B* − *Y*.

We know

$$\begin{array}{l} \begin{array}{c} M_{1}\left[u,r\right]=V\left(k+1,X_{1}+\left\{A_{u}\right\},Y+\left\{B_{r}\right\},w_{1}+1\right)\cdot P_{u,r}+\\ \;V\left(k+1,X_{1}+\left\{A_{u}\right\},Y+\left\{B_{r}\right\},w_{1}\right)\cdot \left(1-P_{u,r}\right)\\ M_{2}\left[u,r\right]=V\left(k+1,X_{2}+\left\{A_{u}\right\},Y+\left\{B_{r}\right\},w_{2}+1\right)\cdot P_{u,r}+\\ \;V\left(k+1,X_{2}+\left\{A_{u}\right\},Y+\left\{B_{r}\right\},w_{2}\right)\cdot \left(1-P_{u,r}\right) \end{array} \end{array}$$

By induction hypothesis, it follows that *M*_1_[*u, r*] ≥ *M*_2_[*u, r*].

Now, let σ denote the equilibrium strategy at *H*_2_ that only selects the top *T* − *k* unmatched players to play (Such a strategy exists according to Claim 2). Note that σ is also a legal strategy at *H*_1_. Let μ(*H*_1_, σ) and μ(*H*_2_, σ), respectively denote the utility of Team 1 when it applies strategy σ on *H*_1_ and *H*_2_. Then,

$$\begin{array}{l} \begin{array}{ccc} \mu \left(H_{1},\sigma \right) & = & \underset{r:B_{r}\in Y}{\min}\underset{u:A_{u}\in A-X_{1}}{\sum }\sigma \left(A_{u}\right)\cdot M_{1}\left(u,r\right),\\ \mu \left(H_{2},\sigma \right) & = & \underset{r:B_{r}\in Y}{\min}\underset{u:A_{u}\in A-X_{2}}{\sum }\sigma \left(A_{u}\right)\cdot M_{2}\left(u,r\right). \end{array} \end{array}$$

From the inequality *M*_1_(*u, r*) ≥ *M*_2_(*u, r*), we get μ(*H*_1_, σ) ≥ μ(*H*_2_, σ). Moreover, we also have *V*(*H*_1_) ≥ μ(*H*_1_, σ) and *V*(*H*_2_) = μ(*H*_2_, σ) (the equality is since that σ is the equilibrium strategy on *H*_2_). Together, *V*(*H*_1_) ≥ *V*(*H*_2_).    □

Finally, Claim 2 of Lemma 3 implies Theorem 2 (1).

### 2.6. Non-transitive Strength

**Definition 4**. *A player is said to be* weakest, *if it is weaker than all its teammates; and is said to be* dominated, *if it has 0 probability to win against any player in the opponent team*.

Assume that the utility function is monotone. In the previous section, we show that if there are redundant players in Team 1 and if the strength of players of Team 1 are transitive, then there is a SPE strategy for Team 1 which does not select the weakest player. In other words, Team 1 can abandon the weakest one without decreasing its utility. In this section, we show that the transitivity is essential for this to hold. We start by the following claim.

**Claim 2**. *Suppose that Team 1 has redundant players, and some player A_u_ in Team 1 is weaker than all its teammate, and yet the players in Team 1 are not transitive. Then, Team 1 might decrease its utility by abandoning A_u_*.

This is somewhat counterintuitive; it might be intuitive that the weakest player has no chance to participate in any match since one can always replace him by a better teammate and increase utility.

Perhaps even more surprisingly, we have the following claim:

**Claim 3**. *Suppose that Team 1 has redundant players, and some player A_u_ in Team 1 is dominated by the other team (i.e., has no chance to win at all), and the players in Team 1 are not transitive. Then, Team 1 might decrease its utility by abandoning the dominated player A_u_*.

The above claims confirm that, the weakest player or even dominated player could help its team.

We would now like to state the organization of the remainder of the section. In subsection 2.6.1, we give examples that verify Claim 3, and we briefly explain the reason why we need dominated players. In subsection 2.6.2, we identify a special case where the weakest player can be abandoned without changing the utility. In subsection 2.6.3, we consider the optimal number of dominated players that we may need to achieve maximum utility. In subsection 2.6.4, we discuss the limitations of the dominated players.

#### 2.6.1. Dominated Teammates Can Be Helpful

Let *V*(*T, P, U*) denote the value of game *G*(*T, P, U*). Let P^*^ denote the sub-matrix of *P* by deleting the last row (thus *G*(T, P^*^, *U*) is the game where Team 1 has abandoned *A*_*m*_).

**Example 2**. *Let n* = *m* = 3, *T* = 2, *U* = *U*_*E*_ *(recall that U*_*E*_(*t*) = *t* − *T*/2). $P=\left(\begin{array}{ccc} 1 & 0 & 0\\ 0 & 1 & 0\\ 0 & 0 & 0 \end{array}\right)$.

In Example 2, there are redundant players and the players in each team are not transitive. Besides, *A*_3_ is a dominated player. We argue the follows: (I) If *A*_3_ is abandoned, Team 2 can win both rounds and hence *V*(T, P^*^, *U*) = −1. (II) If *A*_3_ is in the team, Team 2 cannot win both rounds with certainty and that means *V*(*T, P, U*) > −1. Combining (I) and (II), we get *V*(*T, P, U*) > *V*(T, P^*^, *U*), which implies Claim 3.

Proof of (i): If *A*_3_ is abandoned, Team 2 can play as follows. It chooses *B*_3_ to win the first round. If *B*_3_ defeated *A*_1_, it chooses *B*_1_ in the second round to beat *A*_2_; otherwise, it chooses *B*_2_ in the second round to beat *A*_1_.    □

Proof of (ii): If Team 2 wants to win with certainty in both rounds, it must select *B*_3_ to play the first round. However, if Team 1 selects the dominated player *A*_3_ to play the first round, Team 2 cannot win the second round with certainty anymore.    □

From this example, we see why a dominated player might be helpful for its team. The reason behind is similar to the horse race story described at the beginning of Tang et al. (2010).

In the next, we give one more example. It gives, to our best knowledge, the largest decrease of the value of the game by abandoning a dominated player.

**Example 3**. *Let m* = 4, *n* = *T* = 3. *Let U* = *U*_*E*_ *or U* = *U*_*M*_. *Let* $P=\left(\begin{array}{ccc} 1 & 0 & 0\\ 0 & 1 & 0\\ 0 & 0 & 1\\ 0 & 0 & 0 \end{array}\right)$.

According the method shown in subsection 2.3, we can compute that^4^

$$\begin{array}{l} V\left(T,P,U_{M}\right)=0;V\left(T,P^{*},U_{M}\right)=-2/3;\\ V\left(T,P,U_{E}\right)=-1/2;V\left(T,P^{*},U_{E}\right)=-1/2. \end{array}$$

So, for the game *G*(*T, P, U*_*M*_), we will lose utility as much as 2/3 if we abandon the dominated player.

In the following we explicitly state a SPE strategy for Team 1. In the first round it selects the dominated player *A*_4_. Without loss of generality, assume that it loses to *B*_1_. In the second round, it selects *A*_2_, *A*_3_ uniformly random. So, there is 1/2 chance that Team 1 wins this round. Furthermore, if Team 1 wins the second round (say *A*_2_ beats *B*_2_) it can also wins the next (let *A*_3_ beat *B*_3_) and thus gets utility 1. By this strategy, there is 1/2 chance to get utility 1 and 1/2 chance to get utility −1, so the expected utility is 0.

However, for the game (*T, P, U*_*E*_), we do not lose any utility by abandoning the dominated player. This is not a coincidence. In fact, this example belongs to a special case where the weakest player can indeed be abandoned. We show this in the next theorem.

#### 2.6.2. A Case Where the Weakest Player Can Be Abandoned

We have seen that the weakest redundant player is useless when the players are transitive (as proved in Theorem 2) but might be useful when the players are not transitive (as shown in the previous subsection). So, the next question is:

**Question 1**. *If the players are not transitive, in what cases can the weakest player be abandoned?*

We get the following result.

**Theorem 3**. *Suppose that Team 1 has redundant players but Team 2 does not. So, m > T and n = T. Moreover, suppose that each player in A*_*T*+1_, …, *A*_*m*_ *is weaker than each player in A*_1_, …, *A*_*T*_. *Let U* = *U*_*E*_. *Then, Team 1 can abandon all the players in A*_*T*+1_, …, *A*_*m*_ *without losing its utility*.

**Remark 1**. *According to Example 3, the claim in Theorem 3 fails when U = U_M_. As a comparison, by recruiting extra dominated players, a team can gain more utility when U = U_M_, but cannot when U = U_E_. This may suggest that U_E_ is more reasonable than U_M_ in team competition.*

*The condition m > n = T is important. If both team got redundant players, the claim in Theorem 3 fails*.

We need the following lemma in proving Theorem 3. It is a technical statement of probability theory.

**Lemma 4**. *Assume that n* = *T* and *A*_*i*_, *B*_*j*_ *are any pair of players from the two teams. Let* $Q_{i,j}^{\sigma }$ denote the probability that *A*_*i*_ meets *B*_*j*_ in the game when Team 2 applies the uniformly random strategy and Team 1 applies some strategy σ. Then, $Q_{i,j}^{\sigma }\leq 1/T.$

Proof. We prove it by induction on *n*. The case *n* = 1 is trivial. Suppose that the lemma holds for *n* − 1, and we now argue that it also holds for *n*. Assume that by applying σ, Team 1 has probability *p* to selects *A*_*i*_ in the first round. Then, the probability that *A*_*i*_ meats *B*_*j*_ in the game is at most $p\frac{1}{n}+\left(1-p\right)\left(1-\frac{1}{n}\right)\cdot \frac{1}{n-1}$ (the term $\frac{1}{n-1}$ is due to the induction hypothesis). Therefore, $Q_{i,j}^{\sigma }\leq p\frac{1}{n}+\left(1-p\right)\frac{1}{n}=\frac{1}{n}=\frac{1}{T}$.     □

Proof of Theorem 3: We call *A*_*T*+1_, …, *A*_*m*_ the weak players. When the weak players are abandoned, there are *T* remaining players for each team. By Theorem 1, the uniformly random strategy is a SPE strategy for Team 2. To prove Theorem 3, the key idea is to show that even if Team 1 is allowed to select the weak player, it will not gain more utility if Team 2 keep using the uniformly random strategy. On the other hand, it is obvious that Team 2 can't gain more utility (when Team 1 is allowed to select more players). Therefore, the value of game does not change when the weak players are allowed to play.

First, we compute the utility U^*^ of Team 1 when this team abandons its weak players. As an application of Lemma 4, for any pair of two players *A*_*i*_, *B*_*j*_ (1 ≤ *i, j* ≤ *T*), they meet with a probability no more than 1/*T*. It follows that this probability equals 1/*T*, as the sum of all these *T*·*T* probabilities equals *T*. Because *A*_*i*_ meets *B*_*j*_ with probability $\frac{1}{T}$ and *A*_*i*_ wins *B*_*j*_ with probability *P*_*i, j*_ when they meet, it follows that the number of rounds *t* that Team 1 wins equals $\sum_{j=1..T}\sum_{i=1..T}\frac{1}{T}P_{i,j}$ in expectation. Therefore,

$$\begin{array}{l} U^{*}=\left(\underset{j=1..T}{\sum }\underset{i=1..T}{\sum }\frac{1}{T}P_{i,j}\right)-\frac{T}{2}. \end{array}$$

We now state a formula of the utility *U*^σ^ of Team 1 when it does not abandon its weak players and it applies some strategy σ against the uniformly random strategy of Team 2. Let *Q*^σ^(*i, j*) be defined as Lemma 4. Similar as above, the number of rounds *t* that Team 1 wins equals $\sum_{j=1..T}\sum_{i=1..m}Q_{i,j}^{\sigma }P_{i,j}$ in expectation. Therefore,

$$\begin{array}{l} U^{\sigma }=\left(\underset{j=1..T}{\sum }\underset{i=1..m}{\sum }Q_{i,j}^{\sigma }P_{i,j}\right)-\frac{T}{2} \end{array}$$

We only need to prove that *U*^σ^ ≤ U^*^, and it reduces to showing that for any fixed *j* in 1..*T*,

(2) $$\begin{array}{r} \underset{i=1..m}{\sum }P_{i,j}Q_{i,j}^{\sigma }\leq \underset{i=1..T}{\sum }\frac{1}{T}P_{i,j} \end{array}$$

To prove (2), consider the following optimization problem:

$$\begin{array}{l} \{\begin{array}{cc} \text{Variables:} & x=\left(x_{1},\ldots ,x_{m}\right)\\ \text{Parameters:} & c=\left(c_{1},\ldots ,c_{m}\right)\\ \text{Guarantee:} & c_{i}\geq c_{i^{\prime }}\left(\forall \left(i,i^{\prime }\right)\text{ such that }i\leq T<i^{\prime }\right)\\ \text{Constraint 1:} & 0\leq x_{i}\leq \frac{1}{T}\left(\forall 1\leq i\leq m\right)\\ \text{Constraint 2:} & \sum_{i=1}^{m}x_{i}=1\\ \text{Objective:} & \max f\left(x\right)=\sum_{i=1}^{m}c_{i}x_{i} \end{array} \end{array}$$

Clearly, *f*(*x*) is maximized at x^*^, where $x_{i}^{*}=\{\begin{array}{cc} \frac{1}{T} & i\leq T\\ 0 & i>T \end{array}$.

Noticing the following facts, we see that inequality (2) is just an application of the above problem.

$$\begin{array}{l} \begin{array}{cc} Q_{i,j}^{\sigma }\leq \frac{1}{T} & \left(\text{Applying Lemma}4\right)\\ \sum_{i=1}^{m}Q_{i,j}^{\sigma }=1 & \text{(According to the definition)}\\ \forall i\leq T<i^{\prime },P_{i,j}\geq P_{i^{\prime },j} & \text{(Since}A_{i^{\prime }}\text{is weaker than}A_{i}\text{)} \end{array} \end{array}$$

                    □

#### 2.6.3. Optimal Number of Dominated Players

Here we study the power of dominated players in another direction. As we see in subsection 2.6.1, by abandoning a redundant dominated player, Team 1 may decrease its utility. In other words, Team 1 may increase its utility by recruiting more dominated players. Note that the utility of Team 1 will not decrease by recruiting more dominated players. However, it is unclear that the utility will strictly increase by doing so. For example, recruiting *T* dominated players is the same as recruiting *T* − 1 players — in any case, if any team uses *T* dominated players in a competition, it gets the lowest utility! So, a natural question is:

**Question 2**. *In order to maximize the expected utility of Team 1 (i.e., the value of the game), how many dominated players should we recruit at least? Is it possible that we need as many as Θ(T) such players?*

The theorem below answers this question.

**Theorem 4**.

1. *Suppose U* = *U*_*E*_. *Recruiting T* − 1 *dominated players can be better than T* − 2, *but recruiting T dominated players is the same as T* − 1. *So, to achieve optimal utility, one may require T* − 1 *dominated players. This number is tight*.
2. *Suppose U = U*_*M*_. *Recruiting* ⌊*T*/2⌋ *dominated players can be better than* ⌊*T*/2⌋ − 1, *but recruiting* ⌊*T*/2⌋ + 1 *dominated players cannot be better than* ⌊*T*/2⌋. *So, to achieve optimal utility, one may require* ⌊*T*/2⌋ *dominated players, and this number is tight as well*.

One direction in these claims are rather trivial; we should never use *T* dominated players when *U* = *U*_*E*_ or ⌊*T*/2⌋ + 1 players when *U* = *U*_*M*_. To prove the other direction, we need to construct some examples in which recruiting *T* − 1 (resp. ⌊*T*/2⌋) could be better than *T* − 2 (resp. ⌊*T*/2⌋−1) when *U* = *U*_*E*_ (resp. *U* = *U*_*M*_). To construct such examples, an intuition is that we should make the current players in Team 1 as weak as possible. Our construction is as follows:

**Example 4**. *T* ≥ 1, *m* = *T, n* = *T* + (*T* − 1), *U* = *U*_*E*_, $P_{i,j}=\{\begin{array}{cc} 1 & i=j\\ 0 & i\neq j \end{array}.$

**Example 5**. *T* ≥ 1, *m* = *T, n* = *T* + ⌊*T*/2⌋, *U* = *U*_*M*_, $P_{i,j}=\{\begin{array}{cc} 1 & i=j\\ 0 & i\neq j \end{array}.$

The following claims together prove Theorem 4.

C1. In Example 4, if Team 1 only recruit *T* − 2 dominated players, it can win no rounds and thus can get utility −*T*/2.
C2. In Example 4, if Team 1 recruit *T* − 1 dominated players, it can win a positive number of rounds in expected and thus gain utility more than −*T*/2.
C3. In Example 5, if Team 1 only recruit ⌊*T*/2⌋ − 1 dominated players, it will always lose at least ⌊*T*/2⌋ + 1 rounds and thus can only get utility −1.
C4. In Example 5, if Team 1 recruit ⌊*T*/2⌋ dominated players, it can sometimes win at least ⌈*T*/2⌉ rounds and thus can gain utility more than −1.

Proof of C1: In this case Team 2 can win all the rounds by playing as follows: in the first *T* − 1 rounds, it selects the players *B*_*T*+1_, …, *B*_2*T* − 1_ to play; and they all win. Then, since Team 1 only has *T* − 2 dominated players, at least one player in *A*_1_, …, *A*_*T*_ has already played, denote it by *A*_*i*_. In the last round, Team 2 select *B*_*i*_ and it definitely wins.     □

Proof of C3: In this case, by applying a strategy similar to C1, Team 2 can win all the first ⌊*T*/2⌋ + 1 rounds.^5^     □

Proof of C2: For convenience, we denote the *T* − 1 dominated players by *A*_*T*+1_, …, *A*_2*T*−1_. We argue that, if Team 1 applies the uniform random strategy (that is, select one unused player in *A*_1_, …, *A*_2*T*−1_ uniformly random in each round), then, Team 2 has no strategy to win all rounds all the time. Suppose to the contrary that Team 2 can do it, it must select a player from *B*_*T*+1_…, *B*_2*T*−1_ to play in the first round; otherwise there is a chance that it loses the first round. Note that, since Team 1 apply the uniform random strategy, there is a chance that Team 1 select a dominated player in the first round. If this happens, Team 2 must again select a player from *B*_*T*+1_…, *B*_2*T*−1_ to play in the second round. Once again, Team 1 might still select a dominated player in the second round. By induction, there is chance that Team 1 select all the dominated players in the first *T* − 1 rounds while Team 2 consumes all its *T* − 1 invincible players in *B*_*T*+1_…, *B*_2*T*−1_. Then, Team 2 cannot win with certainty in the last round.     □

The claim C4 is the most non-trivial. To prove it we first state the following lemma.

**Definition 5**. *For integers a, b, C such that*

(3) $$\begin{array}{r} C\geq 1,0\leq a\leq \left\lceil C/2\right\rceil ,0\leq b\leq \left\lfloor C/2\right\rfloor , \end{array}$$

let $\Gamma_{a,b}^{C}$ denote the following instance of team competition:

$$\begin{array}{l} \begin{array}{c} m=n=\left(C-a\right)+\left(\left\lfloor C/2\right\rfloor -b\right),\\ T=C-a-b,P_{i,j}=\{\begin{array}{cc} 1 & i=j\leq C-a\\ 0 & otherwise \end{array}. \end{array} \end{array}$$

The utility is as follows^6^: if Team 1 wins at least ⌈*C*/2⌉−*a* rounds, it gets utility 1 and Team 2 gets −1; otherwise, Team 1 gets utility −1 and Team 2 gets 1.

**Lemma 5**. *For integers a, b, C satisfying condition (3), Team 1 can win utility larger than* −1 *in the game* $\Gamma_{a,b}^{C}$.

Proof. Consider three cases.

Case 1 *a* = ⌈*C*/2⌉. In this case, Team 1 always get utility 1, and so $\Gamma_{a,b}^{C}$ has value 1, which is larger than −1.
Case 2 *b* = ⌊*C*/2⌋. The game $\Gamma_{a,b}^{C}$ can be restated as follows.
  - *m* = *n* = *C* − *a*, *T* = ⌈*C*/2⌉ − *a*.
    Player *A*_*i*_ can only defeat *B*_*i*_ for *i* in 1..*m*.
    Team 1 gets utility 1 if it wins all the rounds; and −1 otherwise.
    We argue that the uniformly random strategy guarantees Team 1 an expected utility larger than −1. Equivalently speaking, by applying the uniformly random strategy, Team 1 has a chance to win all the rounds. The proof is as follows. In the first round, there is a positive chance that *A*_*i*_ meets *B*_*i*_ for some *i*. Then, in the second round, same thing happens with a positive chance. This could happen for each round. When these coincidences happen, Team 1 wins all the rounds.
Case 3 *a* < ⌈*C*/2⌉ and *b* < ⌊*C*/2⌋.
  We use induction. Assume that $\Gamma_{a+1,b}^{C}$ and $\Gamma_{a,b+1}^{C}$ both have value larger than −1, we argue that so does $\Gamma_{a,b}^{C}$. The following facts follow from the definition of $\Gamma_{a,b}^{C}$.
  Fact 1. If the two teams select *A*_*i*_ and *B*_*i*_ for *i* ≤ *C* − *a* in the first round, it becomes a sub-game that is equivalent to $\Gamma_{a+1,b}^{C}$.
  Fact 2. If the two teams select *A*_*i*_ and *B*_*i*_ for *i* > *C* − *a* in the first round, it becomes a sub-game that is equivalent to $\Gamma_{a,b+1}^{C}$.
    Combining them with the induction hypothesis, we get
Fact 3. If the two teams select players under the same index, it becomes a sub-game whose value is larger than −1.
  The value of $\Gamma_{a,b}^{C}$ is equal to the value of the matrix game *M*, where *M*(*i, j*) indicate the value of the sub-game when Team 1 select *A*_*i*_ and Team 2 select *B*_*j*_ in the first round. Fact 3 implies that all the utilities on the diagonal of matrix *M* are larger than −1. So, by using uniformly random strategy over its players, Team 1 can win a utility larger than −1. Therefore, $\Gamma_{a,b}^{C}$ has value larger than −1.    □

Proof of C4: Let *G* denote the revised game of Example 5, in which Team 1 has recruited ⌊*T*/2⌋ dominated players. We could observe that game *G* is almost the same as $\Gamma_{0,0}^{T}$. To be more specific, when *T* is odd, *G* is exactly $\Gamma_{0,0}^{T}$; when *T* is even, the parameters *m, n, T, P* in *G* and $\Gamma_{0,0}^{T}$ are the same; but the utility *U* is slightly different.

Suppose that the value of *G* is −1. Then, Team 2 has a strategy which guarantees a expected utility −1. It means that Team 2 has a strategy which can always win $\left\lfloor \frac{T}{2}\right\rfloor +1$ or more rounds. When Team 2 applies this strategy, Team 1 can never win $\left\lceil \frac{T}{2}\right\rceil$ rounds. It further implies that the value of $\Gamma_{0,0}^{T}$ is also −1. However, this contradicts with Lemma 5. Therefore, the value of *G* must be larger than −1.    □

#### 2.6.4. Limitations of the Dominated Players

Although the presences of dominated players can affect the value of the game, we conjecture that it will not be too much. A question is then,

**Question 3**. *By abandoning a dominated player, how much value might be lost in the worst case? In other words, how much extra (expected) utility can a team gain by recruiting dominated players?*

According to our simulations, we have the following conjecture that we cannot prove at the moment.

**Conjecture 1**. *If *U* = *U*_*M*_, we can gain at most 2/3 extra (expected) utility (in other words, the value of the game increases by at most 2/3) by recruiting arbitrary number of dominated players. If *U* = *U*_*E*_, we can gain at most 1 extra (expected) utility by recruiting arbitrary number of dominated players*.

#### 2.6.5. Throwing a Match and Discarding a Player

Recall the card game between Alice and Bob in subsection 2.1. We shall point out that, recruiting a dominated player in this context can be thought of applying a cheating action, which is to *throw a match* by not placing any card in that round. In the mentioned card game, if Alice and Bob are not allowed to throw a match, Alice can get expected utility −1/3; if Alice is allowed to throw a match, she can get expected utility 1/3. This can be computed according to the method shown in subsection 2.3. Therefore, throwing a match is profitable if permitted.

It may seem unnatural to let a team throw a match like this. The following alternative cheating action called *discarding*, which may seem more natural, is still profitable for the team.

Discarding is defined as follows. Alice (Team 1) is allowed to discard one of its cards and agrees to lose in that round; however the discarded card is never revealed to Bob (Team 2). By discarding, Alice do not gain one more card at hand, unlike the case of throwing a match.

However, if discarding is allowed, it may still be beneficial. We give an instance in which one may gain extra utility by discarding. Formally, we have the following result.

**Claim 4**. *For every integer K* > 0, *there exists a game G such that V*_*K*_(*G*) > *V*_*K*−1_(*G*), *where V*_*K*_(*G*) *denotes the value of game G in which Team 1 is allowed to discard at most K players*.

**Example 6**. *m* = *K* + 1, *n* = 2*K* + 1, *T* = *K* + 1, *U* = *U*_*E*_, $P_{i,j}=\{\begin{array}{cc} 1 & i=j\\ 0 & i\neq j \end{array}.$

The following claims together imply Claim 4.

C5 In Example 6, if Team 1 is only allowed to discard *K* − 1 times, it cannot win in any round.
C6 In Example 6, if Team 1 is allowed to discard *K* times, it can win some rounds in expectation.

Proof of C5: Suppose that Team 1 is only allowed to use discarding *K* − 1 times. Observer that, for any *i* in 1…*m*, after *A*_*i*_ has played and revealed by Team 1, player *B*_*i*_ becomes invincible that he would win with certainty if he plays in the next rounds. Notice that there are *K* invincible players at beginning (which are *B*_*K*+2_…*B*_2*K*+1_) and Team 1 has only *K* − 1 chances to hide a player by discarding. So, in any round, Team 2 has an invincible player at hand. Therefore, Team 2 can win all the rounds.    □

Proof of C6: Consider the following strategy for Team 1. First, Team 1 randomly chooses an order of the players (say, each order with possibility 1/*m*!), and then randomly chooses exactly *K* of its players so that these players will be discarded while playing. We argue that this strategy guarantees Team 1 to win positive rounds in expectation. It reduces to proving that no strategy of Team 2 can win all the rounds against this strategy. Suppose that Team 2 can do so. In the first round, it must select an invincible player (i.e., a player in *B*_*T*+1_…*B*_*T*+*K*_). Otherwise, there is a chance that it loses this round. And then, we know that there is a chance Team 1 discards in the first round. If this happens, Team 2 must also select an invincible player in the second round. Again, it is possible that Team 1 still discards in this round. Continuing this process we see that, it could happen that, in the first *K* rounds Team 2 use all its *K* invincible players, while Team 1 uses discarding *K* times. Then, Team 2 could lose in the *K* + 1-th round.    □

## 3. Materials and Methods (for a Variant): Take Actions in Turn

We now turn to the aforementioned non-simultaneous variant where *Team 2 sends its player before Team 1 in each round*. As mentioned in section 1, our techniques for solving the original simultaneous variant easily extends to the non-simultaneous variant, and most of our results for the non-simultaneous variant are aligned with the results stated in the previous section; see the small difference in Table 1.

First, consider the easiest case where *n* = *m* = *T*. Recall that *S* denotes the set of all perfect matchings between the *T* players in Team 1 and the *T* players in Team 2 (defined in Lemma 2). A key observation is that no matter what Team 2 does, Team 1 can make sure that the matching result between the two teams equals the one in *S* that benefits Team 1 the most (for a given utility function *U*). Thus a simple SPE can be described easily based on this particular matching (However, we do not declare that there is always an efficient algorithm for computing this perfect matching. For example, we are not aware of any good algorithms for computing it when *U* = *U*_*M*_. Yet there are efficient algorithms for *U* = *U*_*E*_).

**Theorem 5**. *When both teams have no redundant players (i.e., *n* = *m* = *T*), then it is a SPE when Team 1 applies the strategy so that the matching result is the same as the one that benefits Team 1 the most*.

For the case of transitive strength, we have the following result which aligns with Theorem 2.

**Theorem 6**. *Assume monotone utility function. Then, (1) If A*_*m*_ ≤ … ≤ *A*_1_, *Team 1 has a SPE strategy which only selects, in each round, one of the players in A*_1_, …, *A*_*T*_. *(2) If B*_*n*_ ≤ … ≤ *B*_1_, *Team 2 has a SPE strategy which only selects, in each round, one of the players in B*_1_, …, *B*_*T*_. *(Be aware that (1) is not symmetric to (2) for the non-simultaneous variant as Team 2 is no longer symmetric to Team 1.)*

Recall the history classes below Lemma 1 and the terminologies introduced above Lemma 3. In addition, when *H* = (*k, X, Y, w*) is a history class (as in the simultaneous case), let *H*(σ) (σ ∈ *B* − *Y*) denote the history class (in the non-simultaneous case) indicating that Team 2 have sent player σ after arriving at *H*.

Proving Theorem 6 (1) reduces to proving the following lemma which is similar to Lemma 3.

**Lemma 6**. *1. Consider a pair of history classes H*_1_ = (*k, X*_1_, *Y, w*_1_) and *H*_2_ = (*k, X*_2_, *Y, w*_2_), where the top *T* − *k* players of *A* − *X*_1_ and the top *T* − *k* players of *A* − *X*_2_ are the same. If *w*_1_ ≥ *w*_2_, we have *V*(*H*_1_) ≥ *V*(*H*_2_).
*2. Consider a non-terminal history class H*(σ) where *H* = (*k, X, Y, w*) and *k* < *T*. Let *A*_*u*_ be the rank *T* − *k* player in *A* − *X*, and let *A*_*v*_ be any player in *A* − *X* that is not a top *T* − *k* player. Then, for Team 1, selecting *A*_*u*_ is at least as good as selecting *A*_*v*_ to play against σ at *H*(σ).

Our proof of Lemma 6 is analogous to our proof of Lemma 3.

Proof. We prove it by backward induction. For *k* = *T*, claim 1 holds obviously and claim 2 holds naturally. Assume the lemma holds for *k* + 1, we now prove that it also holds for *k*.

*Proof of claim 1*. Clearly, $V\left(H_{1}\right)=\min_{\sigma \in B-Y}V\left(H_{1}\left(\sigma \right)\right)$ and $V\left(H_{2}\right)=\min_{\sigma \in B-Y}V\left(H_{2}\left(\sigma \right)\right)$. Therefore, it reduces to proving that *V*(*H*_1_(σ)) ≥ *V*(*H*_2_(σ)) for any σ that belongs to *B* − *Y*.

Applying claim 2 on *H*_2_(σ), we obtain that there exists a top *T* − *k* player *A*_*u*_ in *A* − *X*_2_ such that

$$\begin{array}{l} \begin{array}{c} V\left(H_{2}\left(\sigma \right)\right)=\underset{a}{\underset{︸}{V\left(\left(k+1,X_{2}+\left\{A_{u}\right\},Y+\left\{\sigma \right\},w_{1}+1\right)\right)}}\cdot P_{u,\sigma }\\ +\underset{b}{\underset{︸}{V\left(\left(k+1,X_{2}+\left\{A_{u}\right\},Y+\left\{\sigma \right\},w_{1}\right)\right)}}\cdot \left(1-P_{u,\sigma }\right) \end{array} \end{array}$$

As the top *T* − *k* players in *A* − *X*_1_, *A* − *X*_2_ are the same, *A*_*u*_ is also a player in *A* − *X*_1_, and thus

$$\begin{array}{l} \begin{array}{cc} V\left(H_{1}\left(\sigma \right)\right)\geq & \underset{a^{\prime }}{\underset{︸}{V\left(\left(k+1,X_{1}+\left\{A_{u}\right\},Y+\left\{\sigma \right\},w_{1}+1\right)\right)}}\cdot P_{u,\sigma }\\ +\underset{b^{\prime }}{\underset{︸}{V\left(\left(k+1,X_{1}+\left\{A_{u}\right\},Y+\left\{\sigma \right\},w_{1}\right)\right)}}\cdot \left(1-P_{u,\sigma }\right) \end{array} \end{array}$$

By the induction hypothesis, *a* = *a*′ and *b* = *b*′. Altogether, *V*(*H*_1_(σ)) ≥ *V*(*H*_2_(σ)).

*Proof of Claim 2*. The utilities of selecting *A*_*u*_ and *A*_*v*_ at the history class *H*(σ) are respectively

$$\begin{array}{ll} V=\underset{a}{\underset{︸}{V\left(\left(k+1,X+\left\{A_{u}\right\},Y+\left\{\sigma \right\},w+1\right)\right)}}\cdot P_{u,\sigma }\\ + & \underset{b}{\underset{︸}{V\left(\left(k+1,X+\left\{A_{u}\right\},Y+\left\{\sigma \right\},w\right)\right)}}\cdot \left(1-P_{u,\sigma }\right) \end{array}$$

$$\begin{array}{ll} V^{\prime }=\underset{a^{\prime }}{\underset{︸}{V\left(\left(k+1,X+\left\{A_{v}\right\},Y+\left\{\sigma \right\},w+1\right)\right)}}\cdot P_{v,\sigma }\\ + & \underset{b^{\prime }}{\underset{︸}{V\left(\left(k+1,X+\left\{A_{v}\right\},Y+\left\{\sigma \right\},w\right)\right)}}\cdot \left(1-P_{v,\sigma }\right) \end{array}$$

Notice that *a* = *a*′ and *b* = *b*′ and *a* ≥ *b* according to the induction hypothesis. Therefore,

$$V-V^{\prime }=a\left(P_{u,\sigma }-P_{v,\sigma }\right)+b\left(P_{v,\sigma }-P_{u,\sigma }\right)=\left(a-b\right)\left(P_{u,\sigma }-P_{v,\sigma }\right)\geq 0.$$

    □

Briefly, (for the current round) it is by the definition that the player *A*_*u*_ with rank *T* − *k* performs better than any player *A*_*v*_ with rank bigger than *T* − *k*, and (for the remaining rounds) the set of the top *T* − (*k* + 1) players are the same regardless of who we choose between *A*_*u*_ and *A*_*v*_. Thus, *A*_*u*_ dominates *A*_*v*_.

Theorem 6 (2) can be proved by a similar argument; we only state a key lemma and omit its proof.

**Lemma 7**. *1. Consider a pair of history classes H*_1_ = (*k, X, Y*_1_, *w*_1_) *and H*_2_ = (*k, X, Y*_2_, *w*_2_), *where the top T* − *k* *players of B* − *Y*_1_ *and the top T* − *k players of B − Y*_2_ *are the same. If w*_1_ ≥ *w*_2_, *we have V*(*H*_1_) ≥ *V*(*H*_2_).
*2. Consider a non-terminal history class H* = (*k, X, Y, w*) *(where k < T). Let B*_*u*_ *be the rank T − k player in B − Y, and let B*_*v*_ *be any player in B* − *Y that is not a top T − k player. Then, for Team 2, selecting B*_*u*_ *is at least as good as selecting B*_*v*_ *in the subsequent (k* + 1)-*th round*.

We now move on to the more challenging case where the strength of the players is not transitive. For this case, our first theorem is analogous to Theorem 3 in that it demonstrates a special condition under which we can abandon some weak players.

**Theorem 7**. *Assume the utility function is monotone.*

1. *Suppose m > T and each player in A*_*T*+1_, …, *A*_*m*_ *is weaker than each player in A*_1_, …, *A*_*T*_. *If n = T, Team 1 can abandon all the players in A*_*T*+1_, …, *A*_*m*_ *without losing its utility. However, if n* > *T*, *abandoning these weaker players may decrease the utility of Team 1*.
2. *Suppose n* > *T and each player in B*_*T*+1_, …, *B*_*n*_ *is weaker than each player in B*_1_, …, *B*_*T*_. *No matter m equals T or not, abandoning the players B*_*T*+1_, …, *B*_*m*_ *may decrease the utility of Team 2*.

According to Theorem 7, when a team has redundant weaker players and its opponent team has no redundant players, whether the weaker players can be abandoned depends on which team takes action first.

Proof of Theorem 7: 1. First, assume *n* = *T*. Among all possible matching results between the *m* players in Team 1 and the *T* players of Team 2 that give Team 1 the highest (expected) utility, there exists a matching result *s* that matches *A*_1_, …, *A*_*T*_ to the *T* players of Team 2 (because *A*_*T*+1_, …, *A*_*m*_ are weaker than *A*_1_, …, *A*_*T*_). Team 1 can gain the same utility (implied by *s*) even if *A*_*T*+1_, …, *A*_*m*_ are abandoned.    □

If *n* > *T* and *U* ∈ {*U*_*E*_, *U*_*m*_}, abandoning the weaker players may decrease the utility of Team 1. We prove this by constructing an example in the following (this is basically Example 2 yet *U* is more general).

**Example 7**. *m* = *n* = 3, *T* = 2. *U* ∈ {*U*_*E*_, *U*_*M*_}. $P=\left(\begin{array}{ccc} 1 & 0 & 0\\ 0 & 1 & 0\\ 0 & 0 & 0 \end{array}\right)$.

For this example, Team 1 can win exactly 1 round and will lose all rounds if *A*_3_ is abandoned.

2. We give two examples to prove this claim (one for *m* = *T* and the other for *m* > *T*).

**Example 8**. *m* = 2, *n* = 3, *T* = 2. *U* ∈ {*U*_*E*_, *U*_*M*_}. $P=\left(\begin{array}{ccc} 0 & 1 & 1\\ 1 & 0 & 1 \end{array}\right)$.

In this example, Team 2 can win exactly 1 round and will lose all rounds if *B*_3_ is abandoned.

**Example 9**. *m* = 4, *n* = 5, *T* = 3. *U* = *U*_*E*_. $P=\left(\begin{array}{ccccc} 1 & 0 & 0 & 1 & 1\\ 0 & 1 & 0 & 1 & 1\\ 0 & 0 & 1 & 1 & 1\\ 0 & 0 & 0 & 1 & 1 \end{array}\right)$.

In this example, Team 2 can win exactly 1 round and will lose all rounds if *B*_4_, *B*_5_ are abandoned.     □

We now study the optimal number of dominated players. The following theorem is a counterpart of Theorem 4. It says that for *U* = *U*_*E*_ we need *T* − 1 in the worst case, and for *U* = *U*_*M*_ we need ⌊*T*/2⌋ in the worst case. Interestingly, the same bounds hold for Team 1 and Team 2 and for the simultaneous case.

**Theorem 8**. *The following hold claims for Team 1 and Team 2*.

1. *Suppose U* = *U*_*E*_. *Recruiting T* − 1 *dominated players can be better than T* − 2, *but recruiting T dominated players is the same as T − 1. So, to achieve optimal utility, one may require T − 1 dominated players. This number is tight*.
2. *Suppose U* = *U*_*M*_. *Recruiting* ⌊*T*/2⌋ *dominated players can be better than* ⌊*T*/2⌋ − 1, *but recruiting* ⌊*T*/2⌋ + 1 *dominated players cannot be better than* ⌊*T*/2⌋. *So, to achieve optimal utility, one may require* ⌊*T*/2⌋ *dominated players, and this number is tight as well*.

Proof. The proof of the two claims on Team 1 is easy and is very similar to the proof of Theorem 4. Recall Example 4 and 5 in the proof of Theorem 4. It can be observed that for Example 4 (where *U* = *U*_*E*_), Team 1 can win nothing when it recruits *T* − 2 dominated players, and can win exactly one round when it recruits *T* − 1 dominated players. This means that it needs *T* − 1 dominated players to achieve the optimum utility (and more than *T* − 1 dominated players is obviously not needed). For Example 5 (where *U* = *U*_*M*_), Team 1 can win nothing when it recruits ⌊*T*/2⌋−1 dominated players, and can win as many as *T* − ⌊*T*/2⌋ rounds when it recruits ⌊*T*/2⌋ dominated players. This means that it needs ⌊*T*/2⌋ dominated players to achieve the optimum utility (and more than ⌊*T*/2⌋ dominated players is clearly not needed).

The proof of the claims on Team 2 is also easy but have to use different examples (Note that these examples are not symmetric to the examples given in Example 4 and 5).

**Example 10**. *T* ≥ 1, *m* = *T*+*T* − 2, *n* = *T, U* = *U*_*E*_, $P_{i,j}=\{\begin{array}{cc} 1 & i=j\\ 0 & i\neq j \end{array}.$

**Example 11**. *T* ≥ 1, *m* = *T* + ⌊*T*/2⌋ − 1, *n* = *T, U* = *U*_*M*_, $P_{i,j}=\{\begin{array}{cc} 1 & i=j\\ 0 & i\neq j \end{array}.$

For Example 10 (where *U* = *U*_*E*_), Team 2 can win nothing when it recruits *T* − 2 dominated players, and can win exactly one round when it recruits *T* − 1 dominated players. Therefore it needs *T* − 1 dominated players to achieve the optimum utility (and more than *T* − 1 dominated players is obviously not needed).

For Example 11 (where *U* = *U*_*M*_), Team 2 can win nothing when it recruits ⌊*T*/2⌋ − 1 dominated players, and can win as many as *T* − ⌊*T*/2⌋ rounds when it recruits ⌊*T*/2⌋ dominated players. Therefore it needs ⌊*T*/2⌋ dominated players to achieve the optimum utility (and more than ⌊*T*/2⌋ is clearly not needed).

                       □

## 4. Discussion

In this paper, we study a novel game-theoretic model of situations where two teams make sequential decisions about which of a set of exhaustible actions to select in each round. These actions can be interpreted as team members, cards in a hand, etc. This model has applications in solving the DMRTA problem we introduced at the beginning of this paper. We present a simple SPE for the case where there are no redundant players or the strength of players is transitive. For the other case, we exhibit evidence that the redundant dominated players cannot be easily discounted in their contribution to team performance, which may appear counterintuitive. We investigate the power of the dominated players in three directions: (1) When do they influence the value of the competition? (2) If additional dominated players can be recruited, how many should be required to attain the maximum utility? (3) How much utility might be lost at most if we abandon them? We obtain several non-trivial results that fully or partially answer these questions. We believe that our results are of particular interests to both designers and players of team competitions.

## Data Availability Statement

The original contributions presented in the study are included in the article/supplementary material, further inquiries can be directed to the corresponding author/s.

## Author Contributions

KJ did the key analysis and experiments, wrote the paper, and generalized several preliminary results obtained during his discussions with the other authors. SC joined KJ in several important discussion, which helped KJ in obtaining many preliminary results. PT joined the discussion later, polished the paper, and especially helped in writing the introduction, including the related works. JP helped in finding some applications and joined after we made our conference version. All authors contributed to the article and approved the submitted version.

## Conflict of Interest

The authors declare that the research was conducted in the absence of any commercial or financial relationships that could be construed as a potential conflict of interest. The reviewer VC declared a shared affiliation, with no collaboration, with one of the authors SC to the handling editor at the time of the review.

## Footnotes

^1^This team competition model was introduced in the conference version of this paper, which was accepted by the 2016 International Conference on Autonomous Agents and Multiagent Systems (Jin et al., 2016) [Yet the conference version did not discuss (1) its application in DMRTA and (2) the react-in-turn variant].

^2^Note that there could be other SPEs. For example, when players in Team 1 always lose, any strategies for the two teams form a SPE.

^3^A pure strategy does NOT mean it determines the entire order of players at the beginning. Instead, it means that at each possible history, some unmatched player will be selected deterministically in the upcoming round. In this way, any mixed strategy is a linear combination of the pure strategies.

^4^The value of $G\left(T,P^{*},U_{M}\right)$ and $G\left(T,P^{*},U_{M}\right)$ can be simply computed according to Theorem 1 since there are no redundant players in these games.

^5^In this case Team 2 can actually win all the *T* rounds.

^6^Here the utility functions for two teams are not identical. However, since it is still a zero-sum game, SPE strategies for the teams exists as before. The requirement that the utility functions are identical is not necessary in our model.
